# HS-SA-Based Precise Modeling of the Aircraft Fuel Center of Gravity Using Sensors Data

**DOI:** 10.3390/s19112457

**Published:** 2019-05-29

**Authors:** Xiaoming Guo, Jing Zhang, Lin Tie, Mingqiang Luo

**Affiliations:** 1 School of Automation Science and Electrical Engineering, Beihang University, Beijing 100191, China; xmguo@buaa.edu.cn (X.G.); tielin@buaa.edu.cn (L.T.); 2 School of Aeronautic Science and Engineering, Beihang University, Beijing 100191, China; luomingqiang@buaa.edu.cn

**Keywords:** center of gravity, multidimensional interpolation, heuristic search, simulated annealing, sensor data

## Abstract

The traditional modeling methods of aircraft fuel center of gravity (CG) based on sensor data have some disadvantages, such as large data storage requirements and low computational efficiency. In this article, a novel hybrid heuristic search-simulated annealing (HS-SA) algorithm is used to reduce the data storage requirements and improve the efficiency of the established models based on sensor data. First, a fuel CG model is established based on the multidimensional interpolation of flight sensors and fuel tank data, which can accurately reflect the nonlinear characteristics of the problem and reduce the data storage needs. Then, the calculation nodes are reasonably selected and optimized based on the proposed HS-SA algorithm to improve the precision of the model of the aircraft fuel CG. The established model of the fuel CG has obvious advantages over traditional methods in improving the temporal efficiency and meeting the storage requirements for sensor data in actual flights. Finally, detailed simulations are conducted based on more than 16,000 sets of sensor data, and the results demonstrate the effectiveness of the proposed HS-SA algorithm.

## 1. Introduction

The center of gravity (CG) is a very important design parameter for aircrafts because it directly affects aircraft performance [1] and even flight safety [2,3]. Additionally, the CG directly affects the stability and maneuverability of aircraft [4,5,6]. During flight, the CG of an aircraft is affected by multiple factors, such as fuel consumption, weapon launching, and heavy object launching [7,8]. The CG position will constantly change and requires accurate online estimation. Therefore, a CG calculation model must give accurate results in real time based on the data from aircraft sensors.

The commonly used CG calculation methods are based on the definition of the CG and the basic zero fuel weight of the aircraft, the zero fuel CG of the aircraft, and the fuel weight of each fuel tank obtained by sensors. The advantages of this method based on weight calculations are its simplicity and versatility [9,10,11]. Notably, this approach can be applied to all types of aircraft, but the calculation requires a large set of accurate sensor data regarding fuel weights. However, the calculation accuracy of this traditional method is affected by various factors [12,13,14], such as inevitable inherent errors in the data measurement and calculation processes. The collection of large-scale fuel and aircraft attitude datasets using sensors not only leads to the consumption of storage and computational resources, but also affects the real-time performance of CG estimations [15,16,17]. Therefore, in practical engineering applications, it is necessary to establish a rapid calculation model for the CG of an aircraft with a distributed fuel system and a limited data storage capacity that satisfies certain accuracy requirements. Under the premise of ensuring the accuracy of CG estimation, the calculation model should considerably reduce the consumption of computational resources and effectively improve the estimation efficiency of the CG of an aircraft with a distributed fuel system compared to traditional methods.

An emerging CG online calculation method based on neural networks uses sensor data from flight experiments or wind tunnel experiments as training samples. The neural network algorithm is used to establish the mapping relationships between the aircraft CG and flight parameters [18,19]. Additionally, offline training models and online parameter data from actual flights are used to calculate the estimated CG position. However, as a new research approach, this method is only applicable under specific flight conditions, requires a large and accurate test data set, and consumes a large quantity of computational resources.

The research in this paper focuses on overcoming the shortcomings of those methods, including sensor data storage and low computational efficiency limitations. A novel heuristic search-simulated annealing (HS-SA) algorithm is proposed to establish and optimize the fuel CG model. Based on more than 16,000 sets of sensor data, including the flight attitudes and fuel weights obtained through actual experiments, a cubic spline interpolation method is applied to rapidly calculate the CG of fuel tanks and aircraft. Then, through the reasonable selection and optimization of these nodes based on HS-SA, the precise model achieves the objectives of low data storage and high CG calculation accuracy, which improves the efficiency of the CG model.

## 2. Modeling Scheme of the Aircraft Fuel CG Based on Sensor Data

### 2.1. Precise Modeling Approach for the CG of Fuel Tanks

We establish a fuel CG calculation model that satisfies both calculation accuracy and speed requirements based on sensor data extraction and an analysis of the fuel quality characteristics considering the calculation requirements of the fuel CG. In the modeling process, the calculation accuracy of the model can be assessed according to the sensor data under common flight conditions and fuel consumption status, and the distribution of the reference status points can be optimized by optimizing the model of fuel quality characteristics.

**Figure 1:**
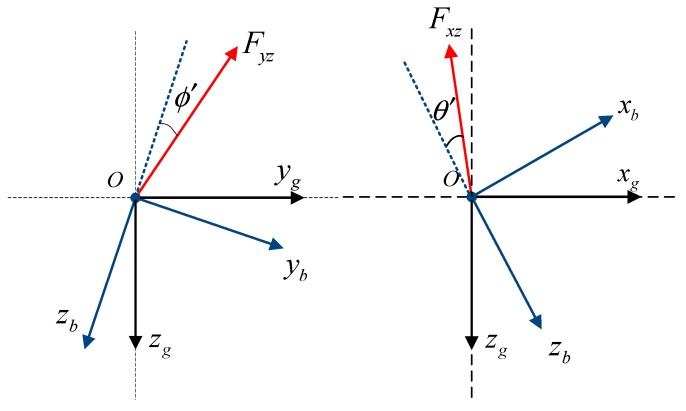
Definition of the angles ($\theta^{\prime }$, $\phi^{\prime }$).

Two angles ($\theta^{\prime }$, $\phi^{\prime }$) related to the acceleration direction are defined. Figure 1 describes the definitions of the angles ($\theta^{\prime }$, $\phi^{\prime }$). Coordinate frames are the Earth-centered inertial frame $ox_{g}y_{g}z_{g}$ and the aircraft-body frame $ox_{b}y_{b}z_{b}$. $F_{yz}$, $F_{xz}$ in Figure 1 represent the components of the net force on the aircraft except gravity in the $oy_{b}z_{b}$ plane and in the $ox_{b}z_{b}$ plane, respectively. Then the angle $\phi^{\prime }$ is defined as the included angle between the force $F_{yz}$ and the negative $oz_{b}$ axis, and its positive value indicating $F_{yz}$ on the left side of the negative $oz_{b}$ axis. Similarly, the angle $\theta^{\prime }$ is defined as the included angle between the force $F_{xz}$ and the negative $oz_{b}$ axis, and its positive value indicating $F_{xz}$ on the downside of the negative $oz_{b}$ axis.

The fuel tank CG rapid calculation model for a single fuel tank can be expressed as $X_{cgfji}||Y_{cgfji}||Z_{cgfji}=f_{j}(W_{F_{j}R_{i}},\theta^{\prime },\phi^{\prime })$. The description of the fuel CG is in the aircraft body coordinate frame. The input parameters include the parameters of the fuel quantity $W_{F_{j}R_{i}}$ and the flight attitude-related parameters $\left(\theta^{\prime },\phi^{\prime }\right)$. For this multivariate nonlinear function $f_{j}(W_{FjRi},\theta^{\prime },\phi^{\prime })$, it is difficult to determine the exact analytical formula. Thus, an interpolation method can be applied to establish the CG calculation model.

**Figure 2:**
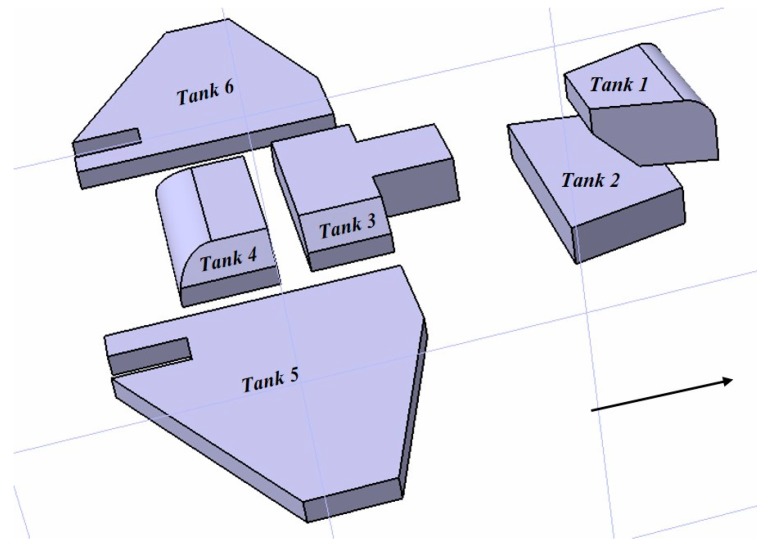
The structure of the aircraft fuel tank.

The modeling method is suitable for aircraft with irregular fuel tank layout. The model object that we selected is an aircraft with an advanced fuel tank layout. The overall layout of the fuel tanks is shown in Figure 2.

**Figure 3:**
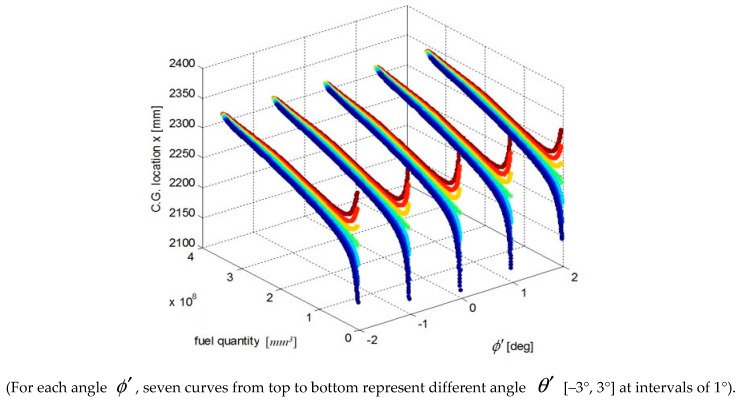
Curve of the fuel tank fuel center $X_{cgfji}$ and input parameters.

**Figure 4:**
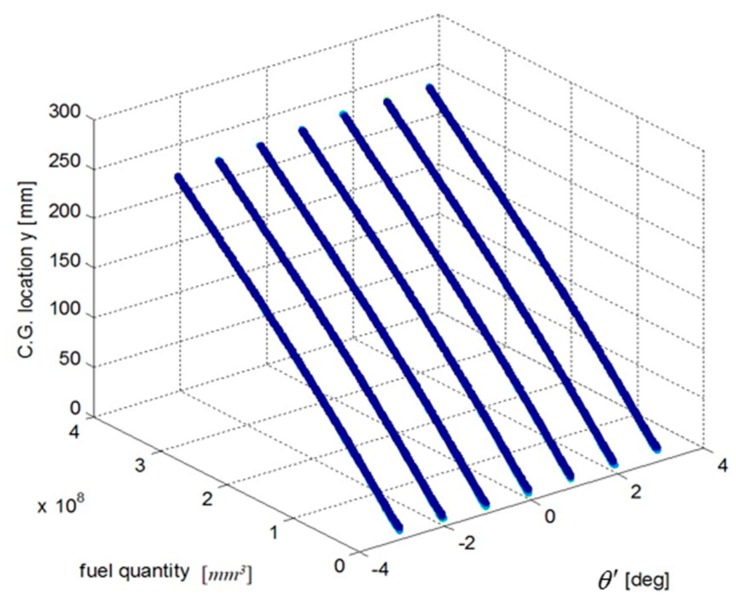
Curve of the fuel tank fuel center $Y_{cgfji}$ and input parameters.

**Figure 5:**
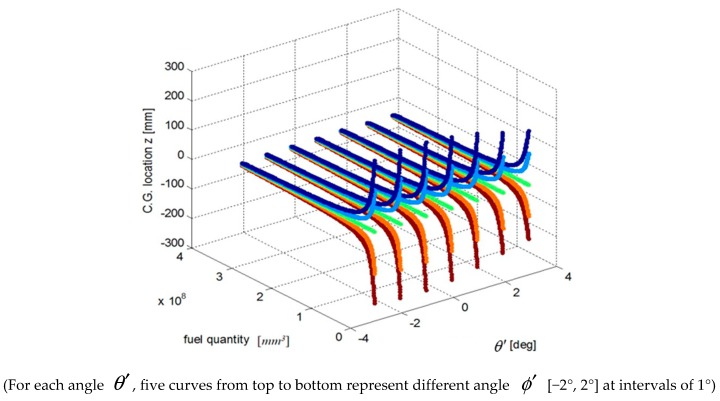
Curve of the fuel tank fuel center $Z_{cgfji}$ and input parameters.

We select a fuel tank based on fuel quality data for the fuel tank and the coordinates of the CG and plot the relationship curves for the fuel CG $X_{cgfji},Y_{cgfji},Z_{cgfji}$ with respect to fuel quantity $W_{F_{j}R_{i}}$, and defined angles ($\theta^{\prime }$, $\phi^{\prime }$), as shown in Figure 3, Figure 4 and Figure 5.

The above input–output relationship curves suggests that the fuel CGs ($X_{cgfji},Y_{cgfji},Z_{cgfji}$) are closely related with fuel quantity $W_{FjRi}$(*mm^3^*) and angle parameters $\left(\theta^{\prime },\phi^{\prime }\right)$. During flight, the fuel CG characteristic curve is a nonlinear function that changes with flight attitude. Therefore, the fuel volume is a key dimension for establishing a multidimensional interpolation model of the CG.

According to this feature of the fuel CG, the cubic spline interpolation is adopted to establish a high-precision model of the fuel CG. This approach can not only extraordinarily reduce the data storage requirements, but also reasonably ensure the accuracy of the CG calculation compared to the traditional table database interpolation approach.

First, a high-precision model of the fuel CG using the cubic spline interpolation method is established. For cubic spline interpolation, the proper selection of calculation nodes is of great importance, because these nodes directly affect the accuracy of the model. Therefore, it is necessary to reasonably select and optimize the nodes based on actual sensor data. The modeling process for the fuel CG calculation model can be described as follows.

(1) The initialization of node selection

Based on the actual sensor data, computational resource availability, and accuracy requirements of the CG calculation model, approximately 300 sets of reference state points are selected as the initial calculation nodes.

(2) Optimization of node selection

The initial model automatically selects an appropriate calculation model and determines the relative error between the results obtained by the selected nodes and the actual data. The next step involves adjusting the selected nodes as needed to optimize the model and improving the calculation accuracy.

(3) The generation of the CG calculation model

Using the final selected calculation nodes, a precise model that meets the relevant accuracy requirements is established, and a corresponding calculation program module is generated.

Taking a single fuel tank as an example, for any fixed flight attitude, the random uniform distribution method can be used to select the initial reference nodes, and a cubic spline interpolation model of the CG of fuel is established. Then, the model is optimized using the HS-SA algorithm.

**Figure 6:**
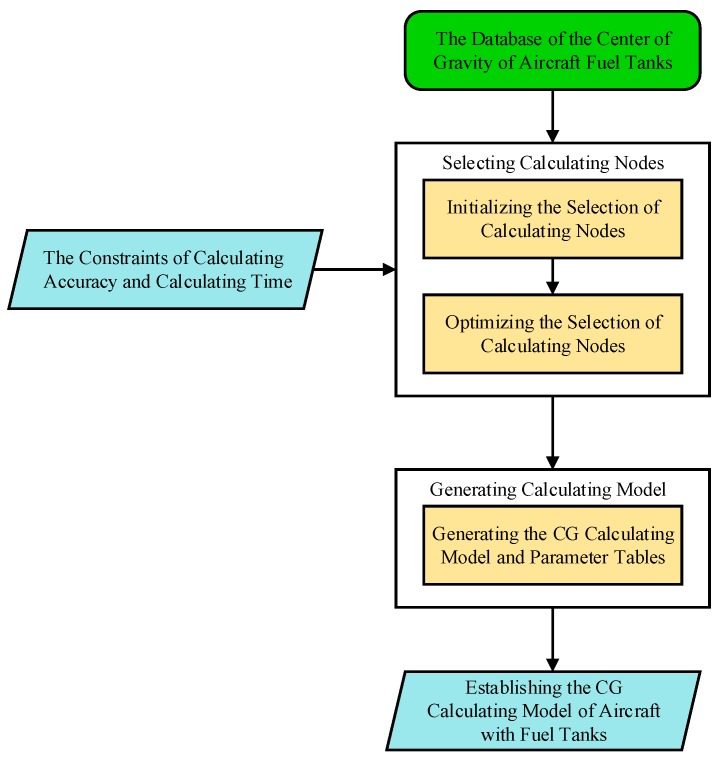
The modeling process of the fuel center of gravity calculation model.

The modeling process is shown in Figure 6.

### 2.2. Realization of a Precise CG Calculation Model

#### 2.2.1. Initialization of Node Selection

If *n* raw fuel quantity data points are available for each dimensional output based on a given fuel tank, the hardware constraint for the data storage quantity is *m*. Because the parameter quantity of each segment in the cubic spline interpolation model is 4, $\left(\frac{m}{4}+1\right)$ reference nodes should be selected from *n*.

The initial selection of the nodes can be based on multiple methods. To simplify the complexity of the initial selection, a random uniform distribution method can be adopted.

Then, a preliminary cubic spline interpolation model can be established. To further improve the accuracy of this model, subsequent optimization steps can be implemented to optimize the selection of nodes.

#### 2.2.2. HS-SA Based Optimization of Nodes

There are many optimization methods for selecting calculation nodes. Further optimization and selection can be performed based on the initial nodes to improve the accuracy and efficiency of the model.

We introduce a new algorithm to optimize the model. This algorithm combines the heuristic search (HS) algorithm [20] with the simulated annealing (SA) algorithm as the HS-SA algorithm.

The SA algorithm is a type of probabilistic jump method with a time-varying search process that eventually tends to zero [21]; therefore, it can effectively avoid local optima and converge to the global optimal solution.

The SA algorithm was first applied by Kirkpatrick et al. [22] in the field of combinatorial optimization. The algorithm is based on probabilistic properties [23] and the similarity between the general combinatorial optimization problem and the annealing phenomenon of solid physical changes. The starting state of the SA algorithm is set to a high initial temperature [24]. The temperature parameter decreases as the process progresses. A random search method is used to find the global optimal value of the objective function in the range of the solution space. Thus, this algorithm eventually yields the global optimal solution and can avoid local optimal solutions [25].

The SA algorithm produces new solutions and accepts new solutions that can be decomposed into four steps.

The first step is to generate a new solution $x_{new}$ in the neighborhood of the current solution x through a random generation function in the solution space. To facilitate the calculation and acceptance steps in the subsequent algorithm and reduce the algorithm time consumption, the method of generating a new solution is based on simple mapping and the current solution. For example, operations such as swapping and replacement are performed for some or all elements constituting the new solution.

The second step is to calculate the difference $\Delta E=E(x_{new})-E(x)$ between the target function value and the new solution $x_{new}$.

The third step is to determine whether to accept the new solution. A default acceptance criterion is the basis for this judgment. The most commonly used acceptance criterion is the Metropolis criterion: if ΔE<0, then the current solution x is replaced by the new solution $x_{new}$; otherwise, the replacement occurs with the probability $e^{-\Delta E/T}$, where *T* is the current temperature.

The fourth step is to accept a new solution. In this case, the current solution is replaced with the new solution. The algorithm only requires the transformed portion of the new solution in the current solution and simultaneously corrects the objective function value to achieve the optimal result. Now, one iteration of the current solution is complete, and the next round of experiments is conducted based on this result. If the new solution is abandoned, the next round of trials continues with the original solution.

Based on the above algorithm, the basic definition of the optimization algorithm for calculation node selection is as follows.

(1) State space and state generation function

In node selection optimization, the state space is described as the set of all possible combinations of $\left(\frac{m}{4}+1\right)$ nodes from *n* data points. For each state, the neighborhood function has multiple options. One of the neighborhood functions that replaces neighboring elements randomly selects a nearby non-calculation data point as a calculation node. If there is more than one such node, one of the non-calculation nodes is randomly selected as the calculation node, and a new state is generated.

(2) Acceptance probability

Using the Monte Carlo criterion, the following relations can be obtained.

(1) $$p=\{\begin{array}{ll} 1 & ,E(x_{new})<E(x_{old})\\ \exp\left[-\frac{E(x_{new})-E(x_{old})}{T}\right] & ,E(x_{new})\geq E(x_{old}) \end{array}$$

(3) Cooling schedule

The cooling schedule T(t) is based on a cooling management table when cooling starts from a relatively high temperature state $T_{0}$ to a relatively low temperature, and the temperature at time *t* can be determined. The SA algorithm can utilize a variety of cooling methods, and two commonly used methods are listed as follows:

(2) $$T(t)=\frac{T_{0}}{\log(1+t)}$$

or

(3) $$T(t)=\frac{T_{0}}{1+t}$$

(4) Initial temperature

The initial temperature $T_{0}$ should be selected considering the optimization efficiency and quality. Because the initial solution is generated in the initial selection of the calculation points, in order to improve the optimization efficiency, the initial temperature cannot be an excessively large value.

(5) Internal loop termination criteria

An inner loop termination criterion is generally used to determine the number of candidate solutions to be generated at a certain temperature. Commonly used criteria include setting a maximum number of steps L and obtaining small consecutive changes in the value of the objective function.

(6) External loop termination criteria

The termination condition of the algorithm may include setting a termination temperature threshold, setting a maximum number of loop iterations, or maintaining the global optimal value for several consecutive steps.

The optimization of node selection based on the HS-SA algorithm is described as follows.

(1) Initialization: Set the initial temperature $T_{0}$, initial solution state x, and number of iterations L to adjust each T value.

(2) Select all *n* data points as nodes, and determine the corresponding cubic spline interpolation function $F_{n}$.

(3) Remove a certain state node $W_{i}$ and calculate the interpolation function and the maximum relative error $E_{\max}$,i relative to $F_{n}$.

(4) From the set of all deletions, the case in which the maximum relative error is the smallest $min\left\{E_{max},i\right\}$ is selected, and the corresponding interpolation function is taken as $F_{n-1}$.

(5) Repeat steps (3) and (4) until $\left(\frac{m}{4}+1\right)$ reference state points remain. And *m* is the constraint of data storage.

The above steps (2)–(5) are the heuristic search part of the HS-SA algorithm. Here, the simulated annealing optimization starts.

(6) Execute steps (7) to (10) for k=1,2,…,L.

(7) Produce a new solution $x_{new}$. $x_{new}$ represents a new set of $\left(\frac{m}{4}+1\right)$ calculation nodes.

(8) Calculate the increment $\Delta E=E(x_{new})-E(x)$, where E(x) describes the maximum relative error of the model.

(9) Accept $x_{new}$ as the new current solution if ΔE < 0; otherwise, accept $x_{new}$ as the new current solution with probability $e^{-\Delta E/T}$.

(10) If the above termination condition is satisfied, the procedure is terminated at this step; otherwise, T is gradually reduced and step (6) is repeated.

The HS-SA algorithm is asymptotically convergent, and optimal node selection can be performed based on the above calculations.

#### 2.2.3. CG Calculation

Using the above nodes for model initialization and data points selection and optimization, the $\left(\frac{m}{4}+1\right)$ interpolation nodes satisfying the accuracy requirements of the model can be determined. Taking $X_{cg}$ as an example, the above method can be used to optimize the nodes for fuel CG calculations and establish a cubic spline interpolation model to form *m*/4 segment intervals. Each segment interval can be described as a cubic polynomial.

$$X_{cg}(W)=a_{xk}+b_{xk}(W-W_{k})+c_{xk}{\left(W-W_{k}\right)}^{2}+d_{xk}{\left(W-W_{k}\right)}^{3},\;k=0,1,\ldots ,\frac{m}{4}$$

For $a_{xk},b_{xk},c_{xk},d_{xk}$, a total of m parameters must be determined according to the second-order continuity equations and boundary conditions of each node. The selection of the boundary conditions must satisfy the not-a-knot boundary condition requirement; that is, the third-order derivative of the boundary node is equal to the third-order derivative of the neighboring node of the boundary node.

The calculation model determined by the above method includes two parts: an interpolation calculation equation and a coefficient data table. The ultimate model uses the coefficient data table instead of the original sensor database, thereby saving a large amount of computational resources.

Denote the output dimension of the coefficient data table as *D*. Using the model obtained with the above method, the required data storage capacity for the interpolation nodes is $3\times \left(\frac{m}{4}+1\right)$, and the data storage occupation of the calculation coefficient is m×D, which effectively reduces the data storage capacity requirement.

The high-precision cubic spline interpolation model for the fuel CG also effectively reduces the complexity of this method. The segmented interpolation model mainly depends on the search operation in the interpolation interval, and the temporal complexity is O(log(n)).

#### 2.2.4. Algorithm Description

According to the above requirements, in the aircraft fuel CG model, the main model steps are as follows.

(1) Parameter initialization. Set the relevant input parameters based on the fuel gravity sensor data for the given tanks and the upper limit of the number of calculation nodes.

(2) Data packets. According to different angle combinations $\left(\theta^{\prime },\;\phi^{\prime }\right)$, the fuel tank sensor data are divided into n groups, and the fuel CG $X_{cg}$ data in different positions are extracted.

(3) Determine the initial calculation nodes. For the sensor data of fuel tanks corresponding to the attitude angle of the i−th group (i=1,2,…,n), a random uniform distribution method is used to determine the initial nodes $W_{0}$, and the corresponding objective function $E_{norm0}$ of these nodes is determined. The objective function calculates the 2-norm error vector of the fuel CG, which is a global performance index that reflects the calculation error.

(4) Optimize the calculation nodes. Based on the initial nodes $W_{0}$, the HS-SA algorithm is used to further optimize the nodes, and the final nodes $W^{*}$ and the objective function $E_{norm}{}^{*}$ are determined through the optimized structure of the inner and outer loops. The inner loop generates a new solution and calculates the increment ΔE of the objective comparison function, and the outer loop avoids local optima based on the temperature reduction coefficient and eventually converges to a global optimum.

(5) Calculate the cubic spline interpolation factor. The cubic spline interpolation coefficients of all output dimensions (i.e., $X_{cg},Y_{cg},Z_{cg}$) are calculated. The final calculation nodes $W^{*}$ output by the HS-SA algorithm are the optimal results.

(6) Save and output the results. Automatically save the final nodes and the interpolation coefficient results. The calculation model can also generate a corresponding Excel file and display the maximum error results for output $X_{cg}$.

**Figure 7:**
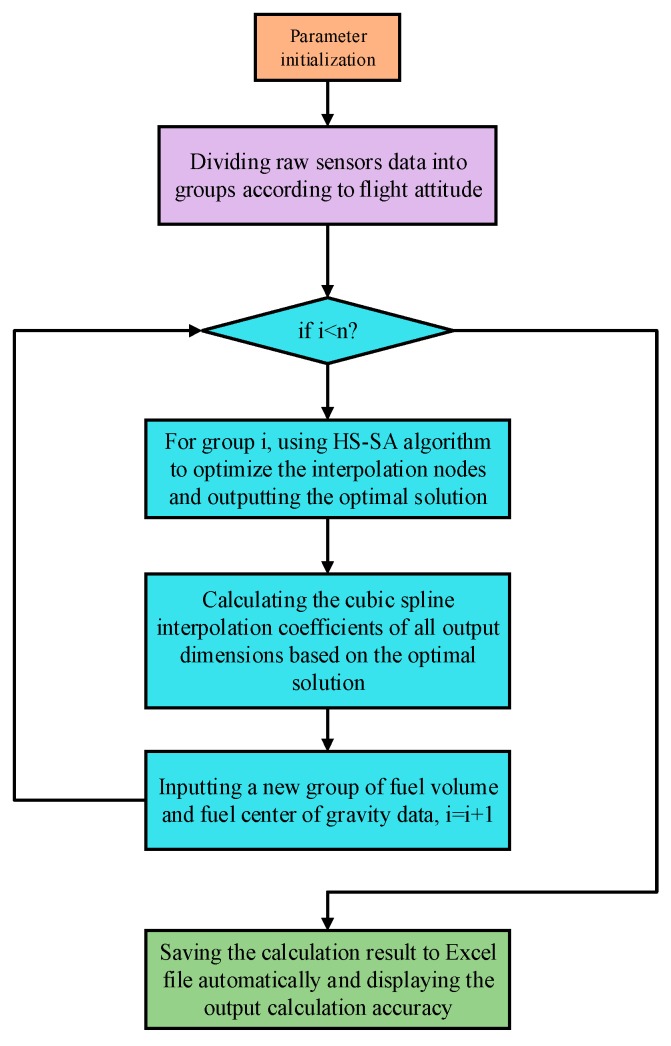
The main program of the calculation model.

Based on the above steps, the structure and flow chart of the main program for calculating the aircraft fuel CG can be summarized as shown in Figure 7.

The main program of the calculation model can automatically optimize the selection of nodes and output the cubic spline interpolation coefficients of all dimensions to meet the requirements of program development.

## 3. Simulation Results and Analysis

### 3.1. Accuracy of the Aircraft Fuel CG Calculation Model

The proposed approach, mentioned above, is only on the basis of fuel weight data and has no special restriction on the tank shape. That is, this modeling approach can handle various tanks with different geometries. 

**Figure 8:**
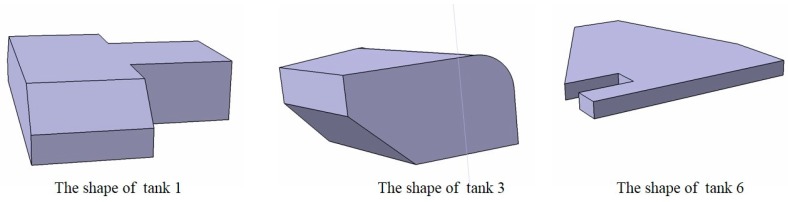
The shape structure of fuel tank 1, tank 3 and tank 6.

In the calculation model of the fuel CG in this scheme, all the actual sensor data for fuel tanks 1 to 6 of a certain aircraft were selected as a test case to verify the accuracy of the aircraft fuel CG calculation model. To verify the effectiveness of the proposed approach, we selected an aircraft with a complex and irregular fuel tank layout as the object. Some geometric shapes of complex tanks are shown in Figure 8.

**Figure 9:**
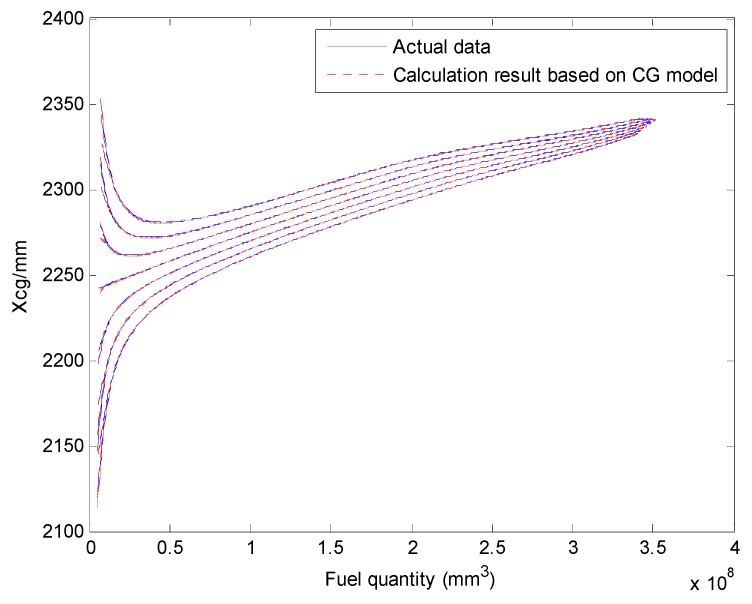
Comparing the $X_{cg}$ results of the fuel CG calculation model with the actual data (tank 1).

**Figure 10:**
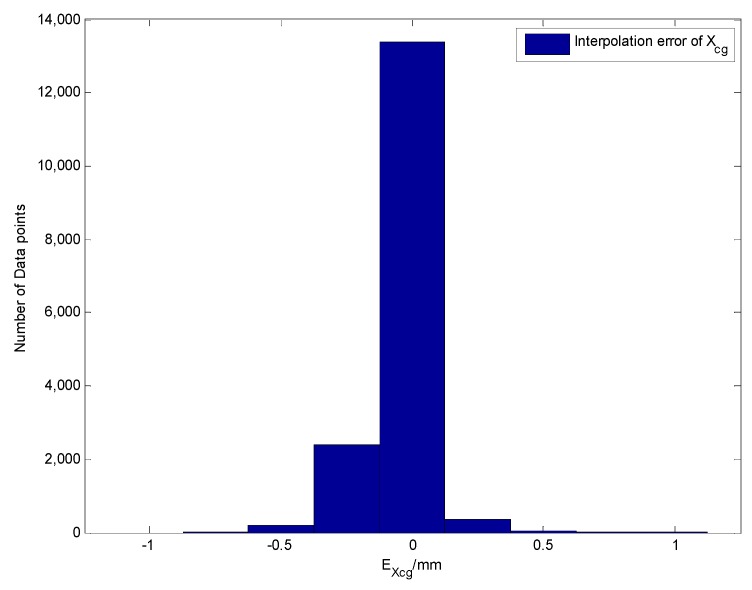
The error distribution (tank 1).

First, for 16,941 sets of actual sensor data, with different attitudes and fuel levels in fuel tank 1, the precision of the output results of the fuel CG calculation model was compared and verified. The shapes of fuel tank 1, tank 3 and tank 6 are irregular, having complex tank geometries, as shown in Figure 8. A comparison curve of the vertical CG of the fuel tanks and the distribution of the calculation error are shown in Figure 9 and Figure 10, respectively.

The verification results indicate that the CG calculation model using this scheme has a high calculation accuracy. The absolute value of the calculation error for 90% or more data nodes is less than 0.5 mm, and the absolute value of the maximum CG error does not exceed 1.5 mm, which satisfies the accuracy requirements of the problem.

**Figure 11:**
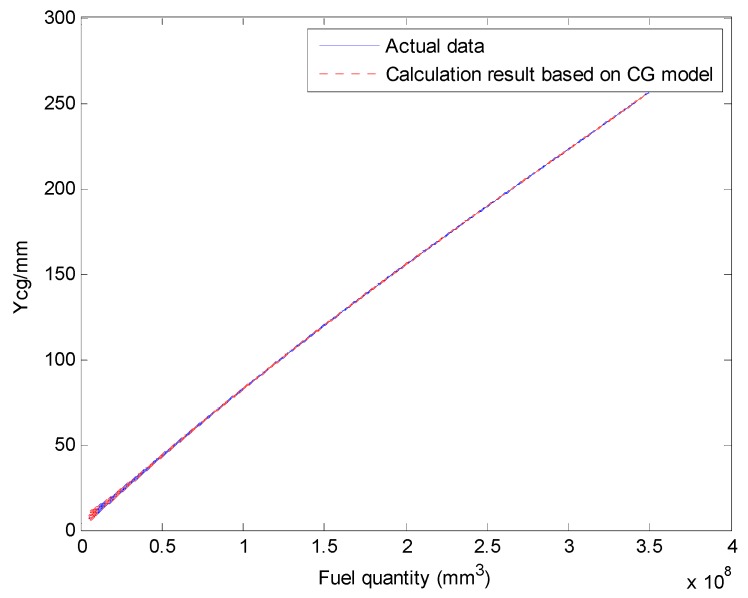
Comparing the $Y_{cg}$ results of the fuel CG calculation model with the actual data (tank 1).

**Figure 12:**
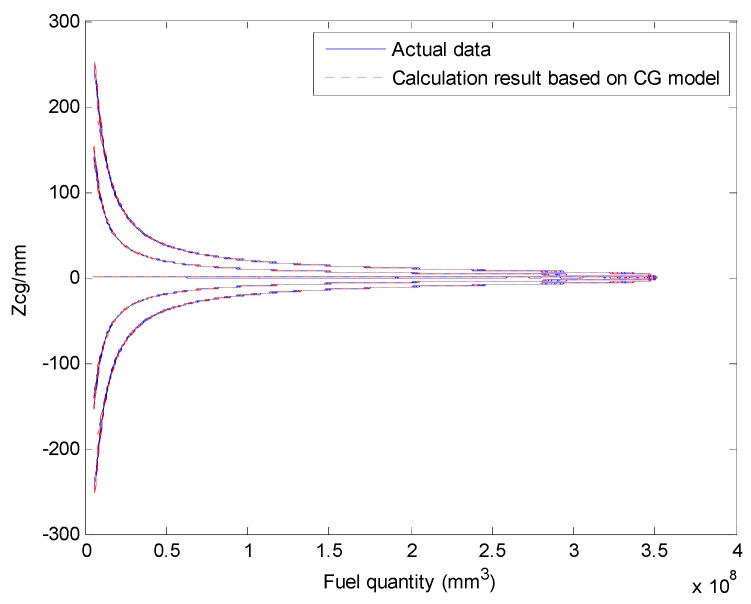
Comparing the $Z_{cg}$ results of the fuel CG calculation model with the actual data (tank 1).

The aircraft fuel CG calculation model results and verification results are shown in Figure 11 and Figure 12.

**Figure 13:**
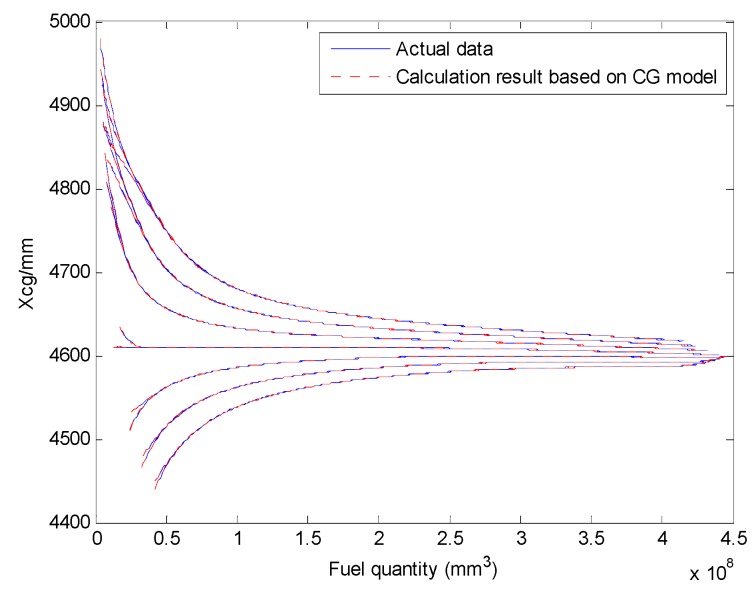
Comparing the $X_{cg}$ results of the fuel CG model with the actual data (tank 3).

**Figure 14:**
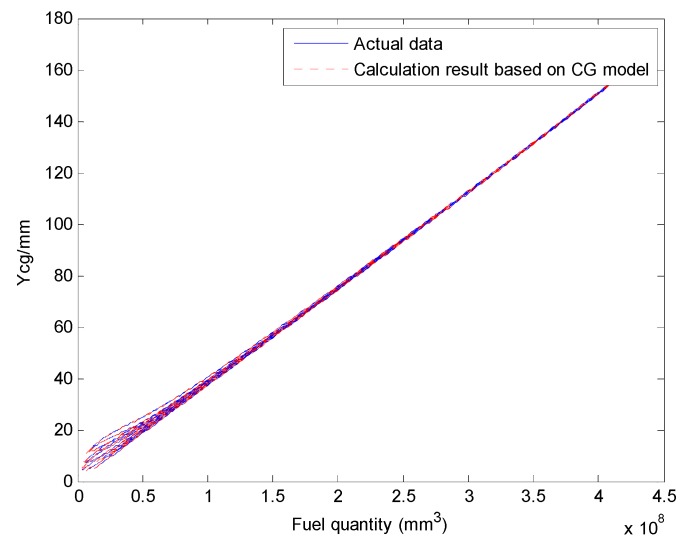
Comparing the $Y_{cg}$ results of the fuel CG calculation model with the actual data (tank 3).

**Figure 15:**
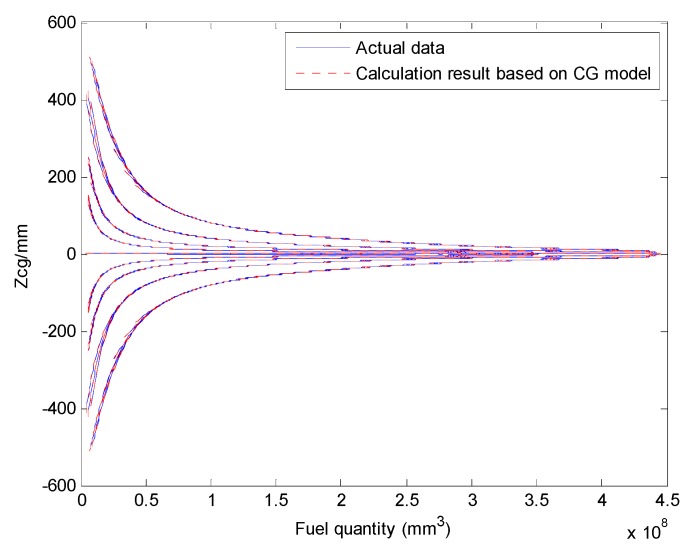
Comparing the $Z_{cg}$ results of the fuel CG calculation model with the actual data (tank 3).

The $X_{cg}$,$Y_{cg}$ and $Z_{cg}$ results for another tank based on the aircraft fuel CG calculation model are shown in Figure 13, Figure 14 and Figure 15. This fuel tank has complex tank geometries, as shown in Figure 8 (2), tank 3.

Based on these irregular tanks, detailed simulations are conducted on the basis of more than 16,000 sets of sensor data. The above test results indicate that the fuel CG calculation model established by the HS-SA algorithm yields a high calculation accuracy for all output dimensions (i.e.,$X_{cg}$,$Y_{cg}$ and $Z_{cg}$), which verifies the effectiveness and advantages of the proposed algorithm.

The aircraft fuel CG calculation model obtained with this solution exhibits high accuracy. Compared with the original data, the average error of the model results for each fuel tank is less than 0.3 mm, and the maximum error is less than 3.6 mm, as shown in Table 1. And the maximum relative error is less than 0.2%, calculated by maximum error and each tank CG.

These values satisfy the accuracy requirements of the CG calculation model. The combined optimization algorithm in this scheme can excessively optimize the calculation nodes and considerably improve the accuracy of the CG calculation model. This advantage is another improvement over traditional methods.

### 3.2. Comparison of Data Storage

Compared with database interpolation, the aircraft fuel CG calculation model can significantly reduce the data storage requirement and effectively decrease the temporal complexity. The total amount of raw sensor data storage for tanks 1 to 6 and the temporal complexity O(log(n)) of each dimension are compared based on the results of the aircraft fuel CG calculation model, as shown in Table 2.

According to the data in the above table, the precise aircraft fuel calculation model can significantly reduce the data storage. The data storage capacity of model only accounts for 6% to 20% of that required for raw data. The data storage capacity of some tanks is reduced by more than 90%, which saves storage resources. Additionally, the model can effectively reduce the temporal complexity of the problem. The efficiency of the calculation time for each dimensional output increases by approximately 30% to 40%.

The results demonstrate that the fuel CG model based on the proposed approach has advantages such as high-precision and small data storage for different types of tank geometries.

## 4. Conclusions

A novel hybrid HS-SA algorithm is proposed for the precise modeling of aircraft fuel CG in this article. First, cubic spline interpolation is applied to establish the CG calculation model and accurately reflect the nonlinear characteristics of the problem. Then, the interpolation nodes are reasonably selected and optimized based on the proposed HS-SA algorithm, which ensures that the CG calculation model has high accuracy. The verification results indicate that the average error of the CG calculations for fuel tanks is less than 0.3 mm and the maximum error is less than 3.6 mm. These results meet the CG calculation accuracy requirements.

Compared with the original database of fuel quality characteristics, the data storage capacity of the aircraft fuel CG calculation model is reduced by more than 80%, and the temporal calculation efficiency is increased by 30% to 40%. These improvements suggest that the established aircraft fuel CG model provides greater accuracy and requires less computational resources. Compared with other methods, this method has outstanding advantages such as a fast calculation speed and small data storage.

The proposed approach mentioned above is only on the basis of fuel weight data and places no special restrictions on the tank shape. Considering the various complex tanks in this manuscript, the cubic spline method and HS-SA algorithm presents excellent performances for both irregular and relatively regular tanks. For different tanks, the verification results indicate that the average error of the CG calculation is less than 0.3 mm and the maximum error is less than 3.6 mm. Therefore, the method has a wide range of applicability and advantages. That is, this modeling approach can handle different types of tank geometries. This method is suitable for tanks with complex geometries. 

In addition, the introduction of a new neural network method in the modeling process may also be our future research direction.

## Figures and Tables

**Table 1:** Calculation errors of the fuel CG model for different tanks.

Tank Number	Average Error/mm	Maximum Error/mm	Maximum Relative Error
1	0.2876	2.7890	<0.13%
2	0.2965	2.9562	<0.14%
3	0.2764	3.5483	<0.18%
4	0.2859	3.2386	<0.18%
5	0.2043	2.3842	<0.15%
6	0.2186	2.2941	<0.14%

**Table 2:** Comparison of CG calculation model results and actual data in terms of the data storage and the temporal complexity.

Tank Number	Data Storage			Temporal Complexity O(log(*n*))	
	Actual Data	Calculation Model	Proportion of Raw Data	Actual Data	Calculation Model
1	203,292	12,390	6.09%	4.2289	2.5441
2	119,292	12,390	10.39%	3.9974	2.5441
3	140,292	12,390	8.83%	4.0679	2.5441
4	143,652	12,390	8.63%	4.0781	2.5441
5	61,332	12,390	20.20%	3.7085	2.5441
6	61,332	12,390	20.20%	3.7085	2.5441
